# Amino Acid Biosignature in Plasma among Ischemic Stroke Subtypes

**DOI:** 10.1155/2019/8480468

**Published:** 2019-01-20

**Authors:** Vânia A. M. Goulart, Marcelo M. Sena, Thiago O. Mendes, Helvécio C. Menezes, Zenilda L. Cardeal, Maria J. N. Paiva, Valéria C. Sandrim, Mauro C. X. Pinto, Rodrigo R. Resende

**Affiliations:** ^1^Department of Biochemistry and Immunology, Federal University of Minas Gerais, Belo Horizonte-MG, Brazil; ^2^Department of Chemistry, Federal University of Minas Gerais, Belo Horizonte-MG, Brazil; ^3^National Institute of Science and Technology in Bioanalytics, Campinas-SP, Brazil; ^4^Institute of Learning and Research Santa Casa de BH, Belo Horizonte-MG, Brazil; ^5^Department of Pharmacology, Federal University of Goiás, Goiânia-GO, Brazil

## Abstract

Ischemic stroke is a neurovascular disorder caused by reduced or blockage of blood flow to the brain, which may permanently affect motor and cognitive abilities. The diagnostic of stroke is performed using imaging technologies, clinical evaluation, and neuropsychological protocols, but no blood test is available yet. In this work, we analyzed amino acid concentrations in blood plasma from poststroke patients in order to identify differences that could characterize the stroke etiology. Plasma concentrations of sixteen amino acids from patients with chronic ischemic stroke (n = 73) and the control group (n = 16) were determined using gas chromatography coupled to mass spectrometry (GC-MS). The concentration data was processed by Partial Least Squares-Discriminant Analysis (PLS-DA) to classify patients with stroke and control. The amino acid analysis generated a first model able to discriminate ischemic stroke patients from control group. Proline was the most important amino acid for classification of the stroke samples in PLS-DA, followed by lysine, phenylalanine, leucine, and glycine, and while higher levels of methionine and alanine were mostly related to the control samples. The second model was able to discriminate the stroke subtypes like atherothrombotic etiology from cardioembolic and lacunar etiologies, with lysine, leucine, and cysteine plasmatic concentrations being the most important metabolites. Our results suggest an amino acid biosignature for patients with chronic stroke in plasma samples, which can be helpful in diagnosis, prognosis, and therapeutics of these patients.

## 1. Introduction

Stroke is a complex neurological syndrome, which involves a sudden abnormality in brain function by interruption of cerebral circulation or bleeding. Each year, approximately 795,000 people in the United States are affected by stroke [1]. About 610,000 of these cases are first attacks and 185,000 are recurrent attacks [1, 2]. Stroke caused by ischemia contributes to 87% of the cases and is triggered by a vascular occlusion, leading to an interruption in oxygen and glucose supply to the brain that affects metabolic processes of the involved region [3, 4]. According to Trial of Org 10172 in Acute Stroke Treatment (TOAST) diagnostic classification system, ischemic stroke can be classified into five subtypes based on its etiology: atherothrombotic, cardioembolic, lacunar, undetermined, and other specific etiologies [5, 6].

Immediately after the ischemic stroke, a cascade of biochemical events promotes the death of brain tissue and subsequent activation of the immune response to area affected [7]. The severity of stroke is directly related to the volume of the lesion, the brain area involved, and the time of the start of treatment[8]. Recognizing the specific cause of strokes, which are etiologically heterogeneous, has important clinical implications [9]. The prognosis and administration of early and long-term strategies to prevent relapse can vary considerably for the different stroke subtypes [10]. For recovery, patients should be treated with specific restorative therapies. Most patients show some improvement, usually during the first 3 to 6 months after the ischemia [11].

The most understanding of how manifestations of neuroplasticity are related to stroke recovery is obtained through multimodal techniques in brain imaging [12]. Currently, the standard techniques for a diagnosis and prognosis of stroke are based on clinical observations and evaluation of neuroimaging [13]. Just as neuroimaging, cardiac evaluation and arterial imaging are used in the diagnosis of stroke, determining its causes and mechanisms of recovery, in the same way molecular features in the form of proteins, RNA, metabolites, lipids, and other biomarkers may also have utility [14].

The metabolomics approach focuses on measurement of the relative concentrations of endogenous small molecules in biofluids, cells, and tissues that characterize changes in metabolism, thus helping to unravel the metabolic state of biological systems [15]. Advances in analytical chemistry together with multivariate statistical methods can allow for the investigation of metabolites as potential biomarkers of various diseases [16–19].

In this study, we compared the amino acid profiles of patients after stroke with healthy subjects and studied different ischemic stroke subtypes. We developed a model to characterize and classify plasma samples of patients from healthy individuals and four stroke subtypes using GC-MS associated with a multivariate method of supervised classification, PLS-DA. Our model determined the amino acid profile that differentiates between healthy individuals from ischemic stroke patients and the amino acids that most contribute to discrimination of each stroke subtypes and controls.

## 2. Methods

### 2.1. Subjects

Human plasma samples were provided by Institute of Education and Research Santa Casa Belo Horizonte, Laboratory of Biomarker. Seventy-three plasma samples from patients diagnosed with one of the ischemic stroke subtypes by magnetic resonance imaging (MRI), based on the TOAST classification system, and being in the chronic phase of the disease (after 7 days of the onset of ischemic stroke diagnostic) were analyzed. We also analyzed 16 plasma samples from healthy subjects (individuals without history of ischemic or hemorrhagic stroke), which were considered the control group. Demographic data are presented in Table 1.

The modified Rankin Scale (mRS) indicates degree of disability of stroke patients (index ranged from 0 to 6). Score 2 designates slight disability or individuals able to look after own affairs without assistance. Score 3 designates moderate disability that will worsen with the increasing of the score value, and the maximum score 6 indicates death. This study was approved by the Ethics Committee in Research of Universidade Federal de Minas Gerais under number 312.840. It was also approved by the Ethics Committee in Research of Santa Casa Misericórdia of Belo Horizonte under number 315.034.

### 2.2. Sample Preparation

The amino acid extraction protocol was adapted from Pinto et al. [20]. Briefly, in order to precipitate proteins, 100*μ*L of plasma samples were dissolved in 900*µ*L of methanol at -10°C. Subsequently, samples were vortexed for 2 minutes followed by centrifugation at 10,000g for 10 minutes at 25°C. The supernatant (100*μ*L) was transferred from each sample into a glass vial and evaporated at room temperature.

Sample derivatization process was performed as described by de Paiva et al. [21]. To each vial containing dried sample was added 15*μ*L of methoxamine solution diluted with 20mg/mL pyridine (Fluka, St. Louis, MO, USA), followed by 35*μ*L N,O-bis (trimethylsilyl) trifluoroacetamide (BSTFA) + Trimethylchlorosilane (TMCS; Fluka, St. Louis, MO, USA). Then, each vial was vortexed for 30s and subjected to 700W of microwave irradiation (Philco, São Paulo, SP, Brazil) for 3 min. After derivatization, the vial was vortexed for 10s. Finally, 1*μ*L of sample was removed with a glass microsyringe (Hamilton, Bonaduz, GR, Switzerland) and manually injected into the chromatograph.

### 2.3. Gas Chromatography Coupled to Mass Spectrometry (GC-MS)

The analyses were performed on a Shimadzu chromatograph, GC-2010/QP-2010 model (Kyoto, Japan), with a high-performance mass analyzer quadrupole. The mass spectrometer was operated in the electron impact mode at 70eV. A fused silica, nonpolar, capillary column (Restek, Bellefont, PA, USA), RTX-5MS model 30m x 0.25mm, id. x 0.25*μ*m), was employed. The temperature program used for oven column was as follows: 80°C for 2min, gradient up to 120°C at a rate of 3°C/min, gradient to 190°C at 8°C/min, gradient up to 300°C at 30°C/min, and then constant 300°C for 3min. The injector was operated at 280°C in the splitless mode for 3min and then at a 1:20 split ratio. Helium gas (99.999%) was used as the carrier gas at a flow rate of 1mL/min. The ion source temperature was 200°C, and the GC-MS interface was kept at 260°C. The analyses were performed in full scan mode, monitoring the mass range of 45-300 m/z, and in the single ion monitoring (SIM) mode for the selection, identification, and quantification of specific ions. Signal acquisition and data processing were performed using the LabSolutions software (Shimadzu, Kyoto, Japan).

### 2.4. Identification and Quantification of Amino Acids

For the method validation, commercial standards of 16 amino acids were used (see Supplementary Materials, Table S1). Standard solutions of each amino acid were prepared and analyzed for construction of calibration curves. Amino acid identification was performed by analyzing the m/z fragments and retention time of specific abundance in the derivatized standard solution, followed by the similarity analysis of the mass spectra with the spectral library. The retention time obtained for each amino acid and the fragments used for quantification are listed in Table S1 (see Supplementary Materials).

### 2.5. Conversion of Peak Areas to Concentration

Calibration curves for each amino acid were built using Excel (Microsoft, USA). For each curve construction, five different concentrations of each standard solution were used. The peak area related to the chosen ions for the measurements was recorded for each concentration and used to build each curve. Through a linear regression, the straight-line equation y = a + bx was estimated, obtaining the slope b and the intercept a. The amino acid concentrations present in the samples were determined in *µ*mol/L using the following equation: peak area+b/a*∗*(1000/molar mass of amino acid)*∗*sample dilution. The correlation coefficients,* r*, were calculated for each analytical curve, so that all curves showed values greater than 0.99.

### 2.6. Statistical Analysis

The Kruskal-Wallis test followed by Dunn's multiple comparison test was used to compare each IS subtype of samples group and control group. To complement the visualization of differences in metabolic profiles, a heatmap was constructed based on the mean amino acid concentration in each subtype of ischemic stroke and controls (see Supplementary Materials).

For supervised classification of IS subtypes and identification of potential biomarkers from 16 amino acids, the multivariate supervised classification method, PLS- DA, was employed [22, 23]. Data were handled with MetaboAnalyst 3.0 web server (https://www.metaboanalyst.ca/) and PLS Toolbox, version 6.5 (Eigenvector Technologies, Manson, WA, USA). For PLS-DA analysis the data were previously autoscaled and leave-one-out cross-validation (LOOCV) was used.

The samples were split into the training and test sets using the Kennard-Stone algorithm[24]. The amino acids with most positive/negative regression coefficients and largest variable importance in the projection (VIP) scores were candidate biomarkers [25]. More details about PLS-DA models and their analytical validation are presented in Supplementary Materials.

## 3. Results

### 3.1. Amino Acid Analysis Distinguishes Patients with Stroke and Control Group

**Figure 1:**
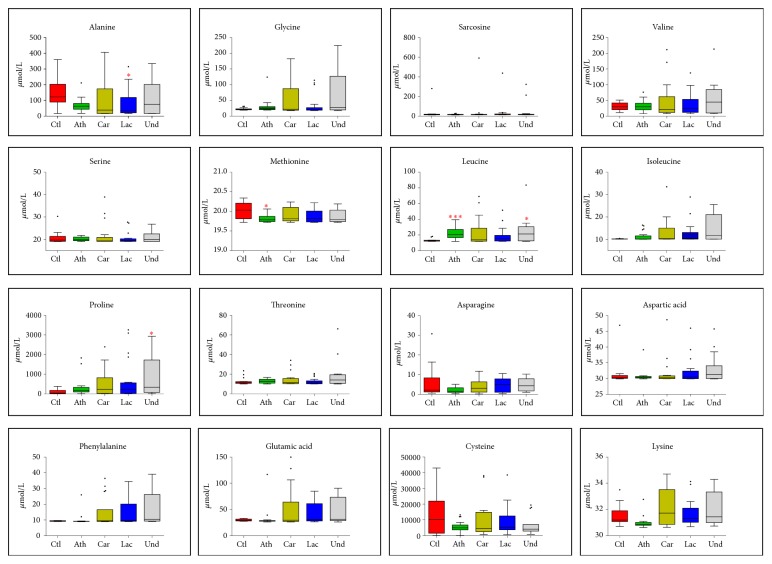
Differences in the amino acid concentrations between stroke subtypes and control individuals. Control individuals (Ctl), atherothrombotic stroke (Ath), cardioembolic stroke (Car), lacunar stroke (Lac), and undetermined stroke (Und). Kruskal-Wallis test followed by Dunn's multiple comparison test: *∗* p ≤ 0.05, *∗∗* p ≤0.01, and *∗∗∗*p ≤ 0.0001.

Plasma amino acid concentrations are kept relatively constant in postabsorptive state of a healthy human; however, circulating amino acid levels change during disease. There is a subtle, but important, change in plasma amino acid concentrations of ischemic stroke patients. The concentrations of 16 amino acids were compared for each subtype of stroke with the control group using the Kruskal-Wallis test followed by Dunn's multiple comparison test to identify statistical differences (Figure 1). Atherothrombotic subtype samples had higher concentrations of Leu and lower concentrations of Met when compared to control. Lacunar subtype samples had lower concentration of Ala when compared to health control. Interestingly, the undetermined subtype samples presented higher concentrations of Leu and Pro than control samples. This fluctuation in amino acid concentration between subtypes and controls can be seen in the heatmap, Figure S1 (see Supplementary Materials).

Based on the plasma concentration of amino acids, we analyzed the biomarker potential of these molecules to discriminate between stroke patients and healthy volunteers by PLS-DA. We built different PLS1-DA models for classifying the plasma samples (see more in Supplementary Materials). The first model was used to classify plasma samples in only two classes, as healthy/control or stroke (independent of the subtype). The other models were developed in order to classify specifically each stroke subtype.

**Figure 2:**
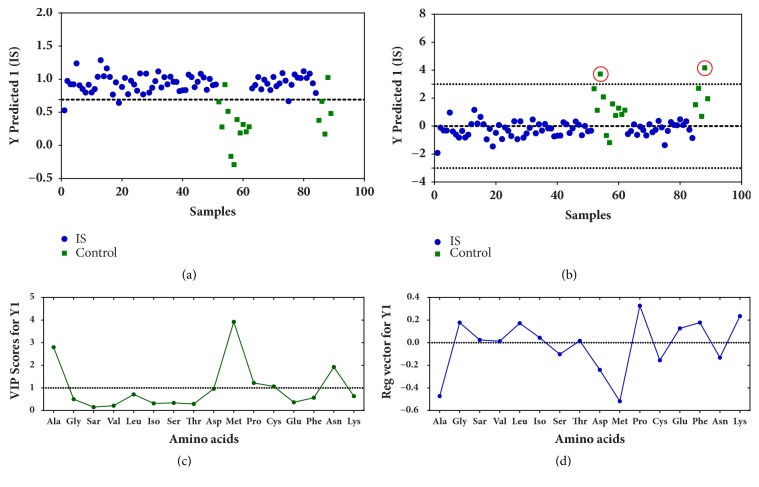
PLS-DA predictions to classify ischemic stroke and control samples (a); studentized residuals for this model at the 99.7% confidence level (b); two samples (red circles) being detected as outliers. Variable importance in the projection (VIP scores) (c) and regression coefficients (d).

The first model (#model 1) was defined considering only two classes, stroke (class 1) and control (class 0) (Figure 2(a)). To obtain a robust model, the data for an independent validation was separated from the external test set. A systematic approach was adopted using the Kennard-Stone algorithm (see Supplementary Materials) to select representative training samples inside each class. Thus, the data were divided into 62 samples for the training set and 27 samples for test set. The best PLS-DA model was selected by LOOCV based on the minimum cross-validation classification error (CVCE).

The best model was built with three latent variables (LV). Model quality can be evaluated through the estimate of figures of merit, such as sensitivity, i.e., the rate of true positives (correct ischemic stroke predictions), and specificity, i.e., the rate of true negatives (correct control predictions). Other parameters used for evaluating the models were cross-validation Q^2^ and area under the receiver operator characteristic (AUROC). For training set, sensitivity was 0.961, and the specificity was 0.909 (Table 2). Nevertheless, only two control samples were incorrectly predicted as ischemic stroke cases, 54 and 88 (circled in red in Figure 2(b)), which can be identified as outliers in a plot of studentized residuals at the 99.7% confidence level. Thus, a global figure of merit can be estimated for the quality of this model, the efficiency rate (EFR) (see Supplementary Materials). The EFR for training and test sets of the developed model was 96.1% and 95.5%, respectively, upon elimination of the two outliers.

Once the reliability of this PLS-DA model was confirmed, the variable statistics (informative vectors) can be used to search for the most discriminant amino acids. The amino acids Met, Ala, Asp, Pro, and Cys presented the most significant VIP scores (Figure 2(c)). The most positive and most negative regression coefficients are the most discriminant ones, directly related to the power of the variables for the classification of classes 1 (ischemic stroke) and 0 (control), respectively. Pro was the most important amino acid for ischemic stroke sample classification, while Met and Ala were the most discriminant ones to classify control samples (Figure 2(d)). Thus, the joint analysis of VIP scores and regression coefficients indicated that Pro, Met, and Ala were the most discriminating amino acids for this model.

**Figure 3:**
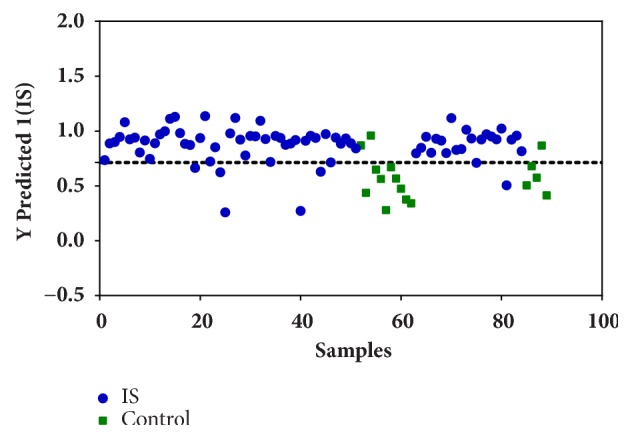
PLS-DA predictions for discriminating ischemic stroke from control samples. This PLS-DA model was built with reduced dataset, containing only the three most discriminant amino acids: Met, Ala, and Pro.

Based on these results, a variable selection was performed, and these three most important amino acids (Met, Ala, and Pro) were chosen as potential biomarkers, in order to build a new PLS-DA model (#model 2). This reduced model was somewhat worse than the full model (Figure 3; Table 2). The regression equation for the reduced model was - 0.20Ala - 0.44Met + 0.53Pro; the negative signs on Ala and Met indicate that plasma samples of healthy individuals have higher levels of these amino acids than ischemic stroke samples. In contrast, the positive sign of Pro indicates that ischemic stroke samples have higher levels of Pro.

### 3.2. Amino Acid Analysis Distinguishes Different Types of Chronic Stroke

To understand the most discriminant amino acids between ischemic stroke subtypes, we tried to develop specific PLS1-DA models (one class against all the other classes) for discriminating each of the four classes: atherothrombotic, cardioembolic, lacunar, and undetermined. Undetermined ischemic stroke was the poorest modeled class, with 36.4% of sensitivity (data not shown). This is coherent with the fact that this is a nonhomogeneous class in terms of etiology causes.

**Figure 4:**
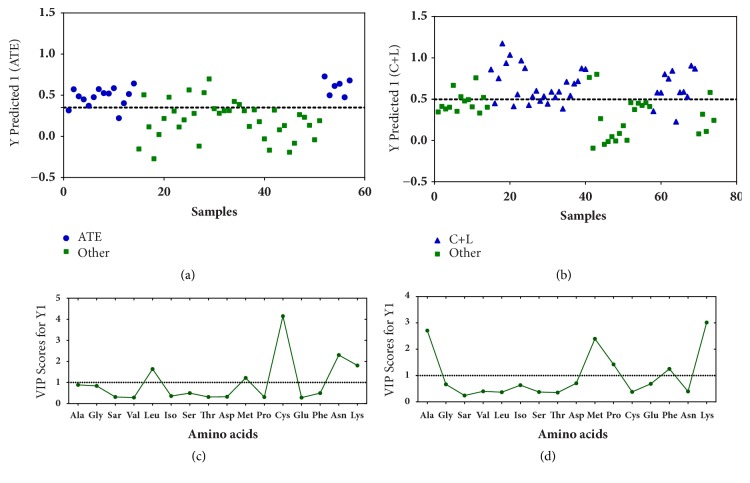
PLS-DA models built with 16 amino acids for classifying ischemic stroke subtypes. Models for discriminating atherothrombotic ischemic stroke from other samples (a) and for cardioembolic plus lacunar ischemic stroke (b). VIP scores (c) for atherothrombotic ischemic stroke. VIP scores (d) for cardioembolic plus lacunar ischemic stroke.

The best-modeled class was atherothrombotic ischemic stroke (Figure 4(a) and Table 3) presenting only two false negatives (out of 14) in the training set and no false negative in the test set. Particularly, cardioembolic and lacunar ischemic stroke samples were poorly discriminated between them, since many samples of one class were predicted as false positive in the other. The regression coefficients for predicting these two classes presented a strong correlation (r=0.9987), indicating their very similar amino acid profiles. Thus, to verify the separation of these two classes compared to others, another model was built including cardioembolic and lacunar ischemic stroke samples in a combined single class. This model showed better results with a sensitivity of 0.833 and a specificity of 0.909 for the test set (Figure 4(b) and Table 3).

The joint analysis of regression coefficients and VIP scores for atherothrombotic ischemic stroke samples (Figures 4(a) and 4(c)) indicated that the most discriminating amino acids were related to the contrast between Leu versus Cys and Lys; i.e., the samples of this subtype were discriminated mainly by higher contents of Leu and lower contents of Cys and Lys. Similarly, by observing the same parameters for the class formed by cardioembolic and lacunar ischemic stroke samples (Figures 4(b) and 4(d)), the main discrimination was related to the contrast between Lys versus Ala and Met.

Using the two first developed PLS models (model #1 and model #2), we can suggest a biosignature for ischemic stroke dependent on Pro, Met, and Ala concentrations. Of the three ischemic stroke subtypes, the best classification was observed for the atherothrombotic subtype, while cardioembolic and lacunar ischemic stroke subtypes were more difficult to distinguish and were modeled together in a single class. This observed similarity may be related to the cardioembolic and lacunar subtypes etiology; both can be caused by thrombosis, besides other possibilities. The lacunar ischemic stroke is related to thrombosis of small arteries, while the cardioembolic one is related to the obstructions of large arteries [26, 27]. We were not able to model undetermined IS subtype due to its intrinsic heterogeneity. Samples of this subtype were predominantly classified in other subtypes, likely because this subtype does not have a common etiology, and its samples may belong to other subtypes [28].

## 4. Discussion

**Figure 5:**
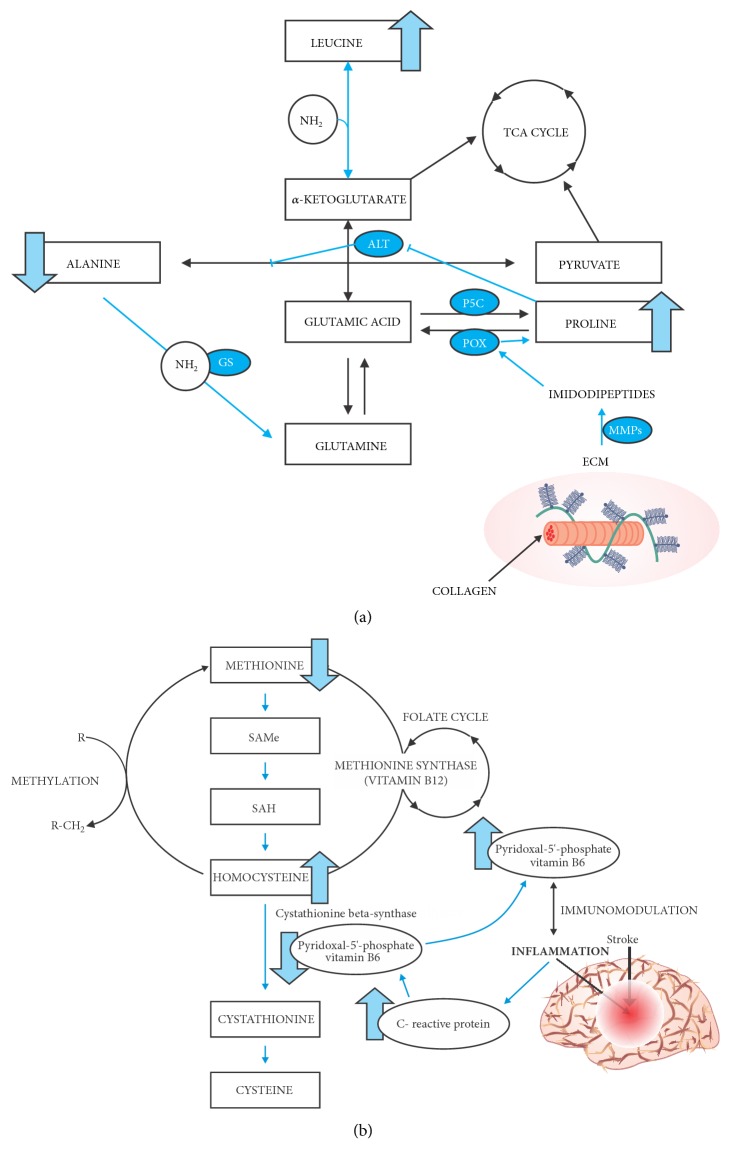
Metabolic pathways activated in chronic ischemic stroke. (a) The increase of proline is promoted by activation of extracellular matrix degradation. High levels of proline can inhibit ALT enzyme and cause alanine concentration reduction. The reduction in alanine concentration can also be related to donation of this amino group for glutamine synthesis, which can be converted to glutamic acid in the neurons and, then, it can be used to increase the levels of leucine. (b) Decrease of methionine concentration due to its conversion to homocysteine; homocysteine increases as a result of the cofactor pyridoxal 5′-phosphate inhibition by inflammatory mediators (C-reactive protein); decrease of pyridoxal 5′-phosphate due to its migration to inflammatory sites. Blue arrows: activated pathways. ECM: extracellular matrix. MMPs: matrix metalloproteinases.

In our study, we observed that amino acids from TCA cycle (Pro, Lys, Ala, and Leu) and the Folate cycle (Met and Cys) are the most important to distinguish chronic ischemic stroke and its subtypes from health volunteers. These amino acids are involved in energy metabolism, in methyl groups generation and DNA synthesis (Figure 5). Recently, Wang and collaborators have demonstrated that tyrosine, lactate, and tryptophan were jointly enabling a high precision (91.7%) to diagnose acute ischemic stroke, within seven days of stroke symptoms [29]. Szpetnar et al. 2017 demonstrated a decrease of proline and a simultaneous increase of glutamate serum level as a marker of acute ischemic stroke; here, we demonstrated an increase in proline as a marker of chronic ischemic stroke [30]. Kimberly et al. 2013 demonstrated that valine, leucine, and isoleucine are reduced in acute ischemic stroke, and here, we observed that leucine is increased in chronic ischemic stroke [31]. Amino acids fluctuations are present in acute and chronic ischemic stroke and it can be used as markers for this pathology.

We highlight the role of Pro as an example: Proline, once oxidized to pyrroline-5-carboxylate (P5C) or its tautomer glutamic-*γ*-semialdehyde, can be interconverted to many other substrates and it is source for carbon exchange between TCA cycle and urea cycle. Furthermore, Pro can be stored in collagen, the most abundant protein by weight in body. As nearly 25% of the residues in collagen are incorporated as Pro, collagen can be a dump as well as a reservoir for Pro. In this context, Pro concentrations may be elevated in plasma from stroke patients as a product of extracellular matrix degradation (mostly collagen) by metalloproteinases activation during brain ischemia (Figure 5(a)). In these low energy-supply cases, Pro metabolic pathway is activated as a cell survival strategy. During nutritional stress, Pro is easily released by extracellular matrix degradation, and this degradation can result in adenosine triphosphate (ATP) production. Proline oxidase (POX), an inner mitochondrial membrane enzyme, catalyzes the released proline to P5C; this process generates electrons that are donated to electron transport chain in mitochondria and generate ATP [32]. There are no reports that indicate how long Pro concentration remains high after a stroke in humans. The use of plasminogen activator (tPA) for the stroke patient treatment can also contribute to the mobilization of Pro from the extracellular matrix. The tPA is an enzyme capable of converting plasminogen to plasmin. Plasmin can degrade several extracellular matrix molecules directly, as well as activating and increasing the metalloproteinases production [33].

The ischemic brain damage is associated with acute and prolonged inflammatory response characterized by activation of the inflammatory resident glial cells as well as the infiltration of leukocytes [34]. In subacute and chronic phases of stroke, infiltrating leukocytes release cytokines and chemokines, and excessive production of reactive oxygen species (ROS) leads to induction/activation of matrix metalloproteinases (especially MMP-9) amplifying the inflammatory response in brain. This process can lead to disruption of the blood brain barrier, cerebral edema, neuronal death, and hemorrhagic transformation [35]. However, many proinflammatory factors play a dual role in early and late stages of stroke. For example, it has been demonstrated that MMP-9 at the beginning of ischemic brain injury operates in a deleterious manner contributing to the enlargement of the lesion, but can promote the regeneration of the brain and neurovascular remodeling at later stages of repair [36]. Activation of matrix metalloproteinases in injury process and repair results in amino acid release, including lysine and proline[32].

Lys is negatively associated with atherothrombotic ischemic stroke and it showed a lower concentration in the samples of this subtype. On the other hand, Lys is positively associated with cardioembolic and lacunar stroke. The role of Lys in stroke has not yet been elucidated, but some studies have shown an important role of this amino acid in reducing ischemic injury and its contribution to angiogenesis process [37]. Based on our results, Lys can be pointed out as an amino acid that can specifically discriminate atherothrombotic ischemic stroke patients from cardioembolic and lacunar ones.

Our results also demonstrated a general decrease in Ala in stroke patient plasma. Karkela and coworkers have analyzed the alanine concentration in cerebrospinal fluid of patients after acute ischemic stroke and found that alanine levels gradually increased within the first four hours, and then, there was a rapid decline [38]. Waagepetersen and coworkers have demonstrated that alanine has an important role as a carrier of nitrogen glutamatergic neurons to astrocytes [39]. It has been found that there is synthesis and release of alanine by glutamatergic neurons while astrocytes are responsible for alanine reuptake. Thus, it has been proposed that this transport through alanine can operate via supplying ammonia to glutamine synthesis in astrocytes and then releasing ammonia through glutaminase reaction inside glutamatergic neurons, thus forming a cycle. In case of ischemic stroke, there is a dysregulation of brain cell processes, so we can infer that the alanine synthesis by glutamatergic neurons is not possible, since adequate concentrations of derivative lactate pyruvate or glucose in neurons are necessary for this synthesis to occur. Another possible explanation for decrease in alanine concentrations in the plasma of stroke patients may be related to proline increase. Furthermore, Shanti et al. have demonstrated that chronic administration of Pro subcutaneously in rats resulted in the inhibition of the activity of enzyme alanine aminotransferase (ALT), cytosolic and mitochondrial, in brain tissues (Figure 5(a)) [40].

Leu concentration also keeps high plasma after stroke, especially in the atherothrombotic subtype. Branched chain amino acids, particularly Leu, play an important role as amino group donors in glutamic acid synthesis in the brain [41]. These amino acids readily cross the blood brain barrier, and Leu can penetrate more rapidly than any other amino acid [42]. In astrocytes, Leu donates the amino group to *α*-ketoglutarate, converting glutamic acid into glutamine in the glial cells (Figure 5(a)). The Leu carbon skeleton is converted to *α*-ketoisocaproate, which, like glutamine, is released into the extracellular medium. The *α*-ketoisocaproate is captured again by neurons through a reverse transaminase reaction, regenerating Leu in a process using glutamate. The newly formed Leu is released to extracellular medium, where it can again be used by astrocytes, completing the Glu-Leu cycle. In neurons, this cycle provides a “buffer” mechanism for glutamate, especially when there is an excess, as is the case during the ischemic cascade in ischemic stroke. This mechanism may explain the increase in Leu concentration in the plasma of patients with ischemic stroke [43].

In our study, we observed that Met concentration was higher in the control group than in all stroke subtypes. The decrease of Met concentrations in stroke patients may reflect the conversion of Met to homocysteine. Methionine is a substrate for the enzyme methionine adenosyltransferase (MAT) being converted to S-adenosylmethionine (SAMe), which is the major donor of methyl groups to coenzymes in the body. The SAMe methylation is a critical step in many proteins stabilization, including myelin [44]. Among other functions, methylation is also important for protection and stabilization of DNA molecules and influences gene transcription. Furthermore, SAMe is involved in polyamines formation, and serotonin and niacinamide metabolism [44, 45]. Methionine also can be used as a precursor of homocysteine (Figure 5(b)). When SAMe transfers its methyl group to an acceptor, S-adenosylhomocysteine is formed. Hydrolysis of S-adenosylhomocysteine by S-adenosylhomocysteine hydrolase (SAH) leads to the formation of homocysteine and adenosine [45]. Although this study did not determine homocysteine levels, an increase of plasmatic homocysteine concentration from patients with occlusive vascular disease or atherothrombotic and lacunar stoke is well-documented[46]. Lindgren and colleagues have found that patients with stroke present increased levels of homocysteine in relation to acute phase of the disease when compared to control group [47].

We found that Cys, a nonessential amino acid, is negatively associated with atherothrombotic stroke. Inflammation process may be related to plasma level decrease of Cys in patients after stroke. Kelly et al. have found high levels of C-reactive protein, which is the primary biomarker of inflammation, in the plasma of patients after ischemic stroke [48]. The contents of this protein were inversely proportional to the levels of pyridoxal 5′-phosphate (PLP), the active form of vitamin B6. PLP acts as a cofactor for a variety of enzymes, including cystathionine *β*-synthase which is responsible for homocysteine conversion into Cys. Low levels of coenzyme PLP in plasma have been associated with their recruitment to inflammatory sites for degradation of tryptophan via the kynurenine pathway, metabolism of sphingolipids, immunomodulatory, and proliferation of immune cells [49]. The decrease in plasma PLP results in decreased Cys and then contributes to the increase of homocysteine levels (Figure 5(b)) [48].

## 5. Conclusion

In this work, we identified amino acid biosignatures in plasma from stroke patients using a GC/MS analysis. The most important amino acids for stroke patient's determination were Pro, Met, and Ala. Although patients are submitted to different environmental factors (e.g., medication use, diet, and risk factors), these amino acids were detected as comprehensive separation between stroke patients and healthy individuals. Other amino acids, Lys, Leu, and Cys, are important to discriminate atherothrombotic subtype from cardioembolic and lacunar stroke. Our results suggest a biosignature for patients with stroke and these amino acids may be considered as biomarkers with great predictive potential.

## Figures and Tables

**Table 1:** Demographic data of the study group.

		**Stroke**			
**Subjects**	**Controls**	**Atherothrombotic**	**Cardioembolic**	**Lacunar**	**Undetermined**
Male	7	13	7	10	8
Female	9	7	11	10	7
**Age (years, mean±SD)**	54.75 (±12.34)	68.70 (±8.09)	62.50 (±12.15)	64.20 (±11.28)	54.93 (±14.50)
**Risk factors**					
Hypertension (%)	68.8	80	83.3	85	67
Diabetes mellitus (%)	0	30	5.6	30	6.7
Alcoholism (%)	18.8	35	27.8	45	26.7
Previous stroke (%)	0	40	33.3	20	26.7
Smoking (%)	31.3	55	44.4	50	33.3
Obesity (%)	18.8	15	11.11	5	13.3
^*a*^ **m** **R** **S** ** ≥ 2 (**%**)**	-	20	44.4	35	26.6

^*a*^mRS: modified Rankin Scale.

**Table 2:** Figures of merit for PLS-DA models to discriminate chronic ischemic stroke from control/healthy plasma samples.

Parameters	Model #1	Model #2
Sensitivity (training set)	0.961	0.902
Specificity (training set)	0.909	0.818
EFR (training set)	0.870*∗*	0.870
Sensitivity (test set)	0.955	0.955
Specificity (test set)	0.800	0.800
EFR (test set)	0.755*∗*	0.720
N° of LV	3	2
Variance in X	66.02%	79.64%
Variance in Y	63.63%	31.62%
Q^2^	0.478	0.176
AUROC	0.800	0.800

*∗* Considering the detection of two control samples as outliers by the studentized y residuals at 95% confidence level, the EFR is 0.961 and 0.955 for the training and test sets, respectively.

**Table 3:** Figures of merit for PLS-DA models for classifying atherothrombotic and cardioembolic plus lacunar ischemic stroke subtypes.

Parameters	Athe*∗*	Card+Lac*∗*
Sensitivity (training set)	0.857	0.769
Specificity (training set)	0.811	0.760
EFR (training set)	0.668	0.529
Sensitivity (test set)	1.000	0.833
Specificity (test set)	0.541	0.909
EFR (test set)	0.541	0.742
N° of LV	4	3
Variance in X	74.77%	63.82%
Variance in Y	27.80%	33.62%
Q^2^	0.359	0.396
AUROC	0.882	0.883

*∗* Athe: atherothrombotic; Card: cardioembolic; Lac: lacunar

## Data Availability

The data used to support the findings of this study are available from the corresponding author upon request.

## Abbreviations

Ala: Alanine
ALT: Alanine aminotransferase
Asp: Asparagine
ATP: Adenosine triphosphate
AUROC: Area under the receiver operator characteristic
Cys: Cysteine
CVCE: Cross-validation classification error
ERF: Efficiency rate
Glu: Glutamic acid
IS: Ischemic stroke
Leu: Leucine
Lys: Lysine
LOOCV: Leave-one-out cross-validation
MAT: Methionine adenosyltransferase
Met: Methionine
MMP-9: Matrix metalloproteinase 9
MRI: Magnetic resonance imaging
mRS: Modified Rankin Scale
P5C: Pyrroline-5-carboxylate
PLP: Pyridoxal 5′-phosphate
Phe: Phenylalanine
Pro: Proline
RNA: Ribonucleic acid
ROS: Reactive oxygen species
SAMe: S-adenosylmethionine
SAH: S-adenosylhomocysteine hydrolase
TOAST: Trial of Org 10172 in Acute Stroke Treatment
Thr: Threonine
tPA: Plasminogen activator
VIP: Variable importance in the projection.

## Supplementary Materials

Supplementary Materials Table  S1. ** Retention time, ions **(**m**/**z**),** and specific abundance for each amino acid. **Figure  S1. ** Heatmap of the ischemic stroke amino acid dataset.** The colors represent the concentration of metabolites. The subtypes of ischemic stroke and controls are represented in horizontal axis and amino acids in vertical axis and are separated using hierarchical clustering (Ward's algorithm), with the dendrogram being scaled to represent the distance between each branch (distance measure: Euclidean). The contrast in the average concentrations of metabolites between the group of patients with stroke and healthy individuals is presented.
