# Supplementary material for: Amino Acid Biosignature in Plasma among Ischemic Stroke Subtypes
Source: Biomed Res Int. 2019 Jan 20;2019:8480468. doi: 10.1155/2019/8480468 (PMC6360633; doi:10.1155/2019/8480468)
Supplement: Supplementary Materials — Table S1. Retention time, ions (m/z), and specific abundance for each amino acid. Figure S1. Heatmap of the ischemic stroke amino acid dataset. The colors represent the concentration of metabolites. The subtypes of ischemic stroke and controls are represented in horizontal axis and amino acids in vertical axis and are separated using hierarchical clustering (Ward's algorithm), with the dendrogram being scaled to represent the distance between each branch (distance measure: Euclidean). The contrast in the average concentrations of metabolites between the group of patients with stroke and healthy individuals is presented. [file 8480468.f1.pdf]

Supplementary tables

Table S1. Retention time, ions (m/z) and specific abundance for each amino acid

Nº	Amino acids	Retention time in minutes*	Identification ions (m/z) and abundance (%)	Quantitation ions (m/z)
1	L-Alanine	8.51±0.03	116(100)+73(53.6)	116
2	L-Glycine	9.35±0.03	102(100)+73(68.4)	102
3	Sarcosine	12.22±0.02	116(100)+73(52.0)	116
4	L-Valine	12.29±0.02	144(100)+73(40.6)	144
5	L-Leucine	14.40±0.02	158(100)+73(42.4)	158
6	L-Isouleucine	15.25±0.02	158(100)+73(50.4)	158
7	L-Serine	17.60±0.02	73(100)+204(75.2)	204
8	L-Threonine	21.83±0.02	73(100)+218(46.0)	218
9	L-Methionine	21.9±0.02	176(100)+128(60.0)	176
10	L-Aspartic acid	20.24±0.02	73(100)+232(68.4)	232
11	L-Proline	21.15±0.02	156(100)+73(53.6)	156
12	L-Cysteine	22.15±0.02	73(100)+220(88.8)	220
13	L-Glutamic acid	22.99±0.03	246(100)+128(32.4)	246
14	L-Phenylalanine	23.00±0.02	218(100)+192(71.2)	218
15	L-Asparagine	23.75±0.03	116(92.40)+231(51.2)	231
16	L-Lysine	24.45±0.03	84(100)+156(55.6)	156

*Mean ± standard deviation (n=10). Adapted from de Paiva *et al*., 2013

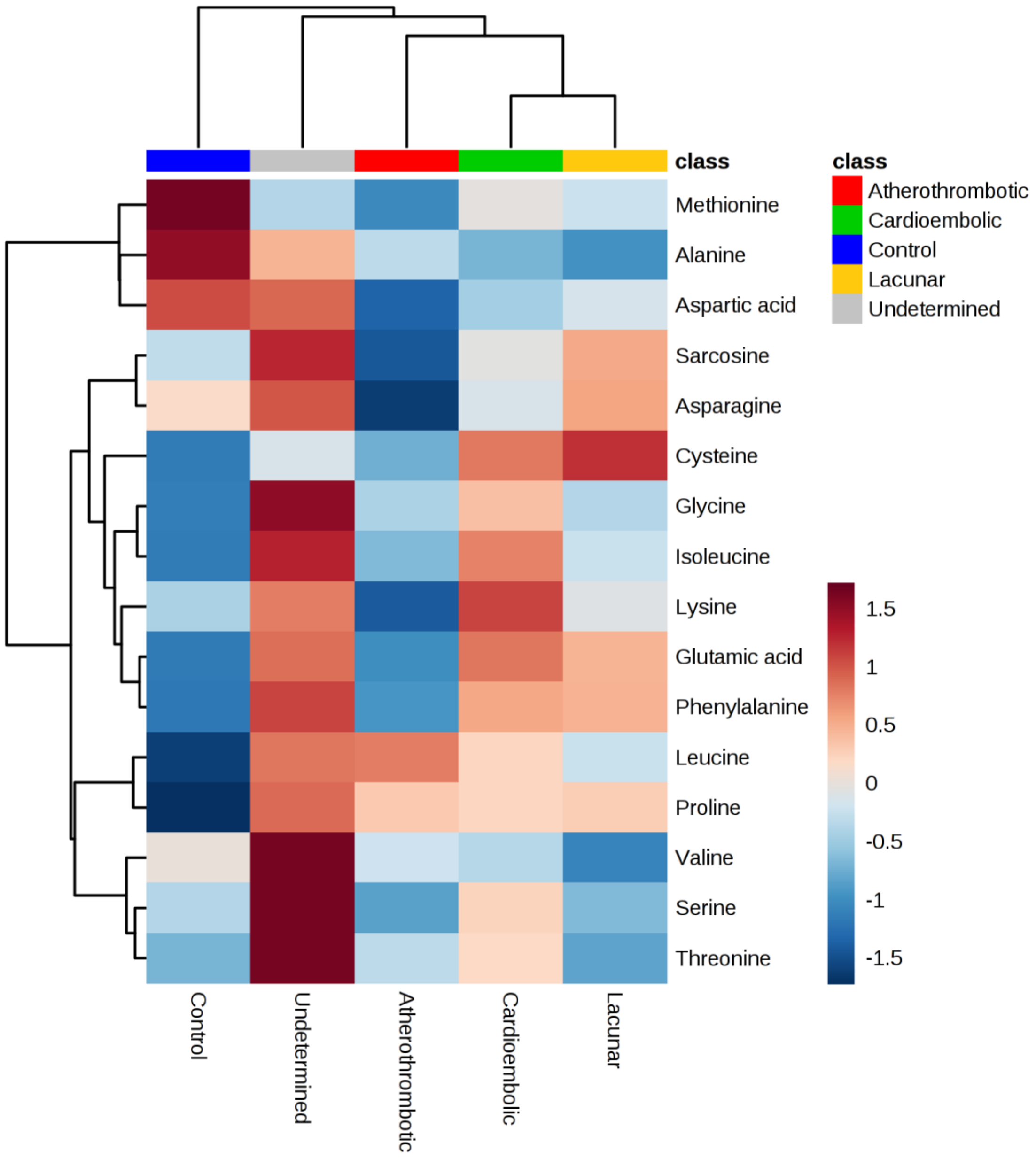

Figure S1. Heatmap of the ischemic stroke amino acid dataset. The colors represent the concentration of metabolites. The subtypes of ischemic stroke and controls are represented in horizontal axis and amino acids in vertical axis; and are separated using hierarchical clustering (Ward's algorithm), with the dendrogram being scaled to represent the distance between each branch (distance measure: Euclidean). The contrast in the average concentrations of metabolites between the group of patients with stroke and healthy individuals are being presented.
